# Formation and Characterization of Irreversible Sediment of Ginseng Extract

**DOI:** 10.3390/foods10112714

**Published:** 2021-11-05

**Authors:** Di Qu, Mei Hua, Jian-Bo Chen, Shan-Shan Li, Lian-Kui Wen, Yin-Shi Sun

**Affiliations:** 1 Institute of Food Science and Engineering, Jilin Agricultural University, Changchun 130118, China; qudi@caas.cn; 2 Institute of Special Animal and Plant Sciences, Chinese Academy of Agricultural Sciences, Changchun 130112, China; huamei@caas.cn (M.H.); chenjianbo00882@126.com (J.-B.C.); lishanshan@caas.cn (S.-S.L.)

**Keywords:** ginseng, botanical beverages, chemicals, sediment, structural characterization

## Abstract

Sediment is a key issue in the beverage industry. This study confirmed that reversible and irreversible sediments were formed during low-temperature storage of ginseng extract. The first 30 days of storage are the critical period for sediment formation. As the time of storage extends, the chemical composition changes. The composition interaction model verified that the cross-linking of protein–pectin, protein–oxalic acid and Ca^2+^–pectin was the main cause of the turbidity of ginseng extract. Based on the characterization of irreversible sediment (IRS), there are typical structures of proteins, polysaccharides and calcium oxalate dihydrate (COD) crystals. Glucose, galacturonic acid, aspartate, glutamic acid, leucine, Ca, K, Al, Mg, Na and Fe are the main monomer components. Effective regulation of these ingredients will greatly help the quality of ginseng beverages.

## 1. Introduction

Botanical functional beverages have been developing rapidly in recent years because of their unique and natural health advantages. Ginseng (*Panax ginseng* C.A.Mey) contains saponins, polysaccharides, amino acids, minerals and other nutrients [1,2]. It has antifatigue, antiaging, antitumor and other effects [3,4,5]. Ginseng is widely used in health food in China, South Korea, Japan and other Asian countries as a medicine-food homologous material. However, ginseng beverages generate sediment during storage, which not only affects the appearance quality of the product but also reduces the unique aroma and flavor of ginseng beverages and causes the loss of functional ingredients. At present, physical methods such as filtration are mainly used to remove this type of sediment, but these methods are not effective enough [6,7]. Therefore, elucidating the formation mechanism of ginseng beverage sediment can provide basic support for the precise regulation of sediment and help improve the quality of ginseng beverages.

Beverage sediment is a key issue of common concern by scholars in the industry, and the formation of beverage sediment has an important relationship with the interaction of chemical components in plants [8]. There were significant differences in chemical composition of different parts and ages of ginseng. Carbohydrates are the most abundant chemical components in ginseng, followed by protein [9]. Part of the sediment can be reheated and reconstituted into reversible sediment (RS), and the irreversible part is called irreversible sediment (IRS), which is similar to the sediment in green tea [10]. The common phenomenon of turbidity in beverages is a result of polyphenol–protein interactions, which bind noncovalently and are reversible in the early stage of sediment formation [11]. The binding between polysaccharides and proteins is another reason for sediment formation. Sediment formation is a spontaneous process promoted by electrostatic interactions, hydrogen bonding and van der Waals forces [12]. The polysaccharide–protein interaction is reversible. When the pH value is lower than the isoelectric point of the protein, the protein is positively charged and can form complex coacid salts. When the pH value is higher than the isoelectric point of the protein, the anionic glycan forms weak electrostatic interactions with the positively charged part of the protein, resulting in the formation of soluble complexes [13,14]. Minerals and organic acids in plants can combine to form insoluble mineral salts [15,16,17]. The irreversible sediment of green tea is mainly the combination of Ca^2+^ and oxalic acid [18]. The crystalline component in the sediment of barberry juice forms from potassium salt [17]. The tartrate in grape juice will be deposited as the temperature decreases during crystallization [19]. In addition, sediment will be deposited with the extension of storage time, and the phenolic substances in mulberry juice will gradually precipitate [20]. Caffeine and proteins in the reversible sediment of green tea are significantly increased, and most of the minerals participate in the formation of a precipitate [21].

The complexation of chemical components in beverages can significantly affect the functional properties of food, such as solubility, surface activity, conformation stability, gel formation ability, emulsification ability, etc. At present, there are few studies on the sediment in ginseng extracts. This article studies ginseng extract stored at low temperature for 0–50 days, analyzes the chemical composition of reversible and irreversible sediments, establishes component interaction model, and reveals the sediment formation mechanism through the structural characterization of IRS. It also provides a theoretical basis for the inhibition of ginseng beverage sediment formation and the retention of functional components.

## 2. Material and Methods

### 2.1. Graphical Scheme of the Approach of the Study

The technology roadmap for the study is as follows:


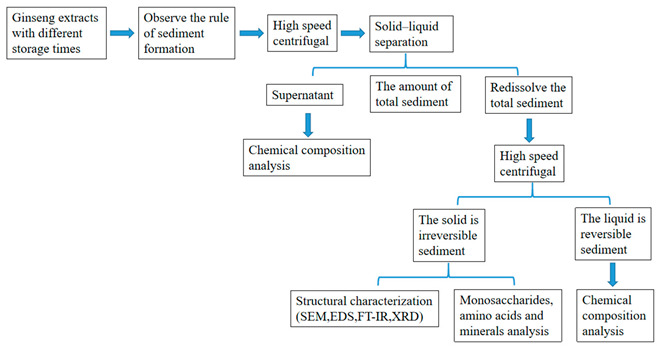


### 2.2. Preparation of a Ginseng Extract

Panax ginseng (five years old) was produced in Fusong County, Jilin Province. After crushing and sieving, 20- to 60-mesh particles and purified water were extracted at 80 °C for 60 min at a mass ratio of 1:10. The purified ginseng extract was filtered with a double-layer 300 filter cloth and centrifuged at 5000 rpm for 15 min. The purified ginseng extract was separated into 50 mL centrifuge tubes and pasteurized (90 °C, 5 min).

### 2.3. Observation of Precipitate Formation

The prepared ginseng extract was stored at 4 °C for 50 days. After centrifugation (10,000 rpm, 15 min), the sediments were described in terms of sediment amount, color and morphology and were evaluated every 10 days. The evaluation team was composed of 9 teachers majoring in food science.

### 2.4. Preparation of RS and IRS

The stored ginseng extract was centrifuged at 10,000 rpm for 15 min, and the supernatant was removed. The total sediment (TS) was diluted to 50 mL with purified water, heated at 70 °C for 20 min, vibrated and then centrifuged (10,000 rpm, 15 min) to separate the precipitates. The liquid part was RS, and the bottom sediment was IRS.

### 2.5. Determination of the Amount of Sediments

The separated total sediment and IRS were dried at 80 °C for 48 h and weighed. The specific procedure was performed according to Nagalakshmi et al. (1984) [22].

### 2.6. Analysis of Chemical Composition

#### 2.6.1. Analysis of Total Sugar, Protein and Total Free Amino Acids

The total sugar content was determined by the phenol–sulfuric acid method. Glucose was used as the reference substance. Then, 0.5 mL of 5% phenol and 2.5 mL of sulfuric acid were added to a 1 mL sample and mixed quickly; the sample was kept at 40 °C for 30 min and then rapidly cooled to room temperature [23]. The optical density (OD) was measured at 490 nm using an EPOCH microplate reader (Bio Tek Instruments, Inc., Highland Park, Winooski, Vermont, USA). The calculation formula is as follows: total sugar content (mg·mL^−1^) = 10.979 × OD_490_ + 0.0059, *R*^2^ = 0.9994.

The protein content was determined by a BCA Protein Quantitative Kit (Sangon Biotech, Shanghai, China).

The content of total free amino acids was determined by ninhydrin colorimetry. Glutamic acid was used as the reference substance. Then, 0.5 mL of buffer solution at pH = 6.81 and 0.5 mL of 20 g/L ninhydrin solution were added to 1 mL of sample and heated in boiling water for 15 min. After cooling, the volume was fixed to 25 mL, and the OD was determined at 570 nm [24] according to the following formula: total free amino acid content (mg·ml^−1^) = 2.012 × OD_570_ − 0.2058, *R*^2^ = 0.9997.

#### 2.6.2. Analysis of Total Saponins

The total saponins were determined by the vanillin–sulfuric acid method. Ginsenoside Re was used as the reference substance. A 40 μL sample was evaporated, and 0.2 mL of 5% vanillin and 1 mL of 72% sulfuric acid were added. The reaction was carried out at 60 °C for 15 min. After cooling, 5 mL of glacial acetic acid was added and mixed evenly. The optical density (OD) was determined at 560 nm. The formula for the calculation was as follows: total saponin content (mg·ml^−1^) = 2.0055 × OD_560_ − 0.001, *R*^2^ = 0.9993.

#### 2.6.3. Analysis of Ginsenosides

The 8 ginsenosides to be tested were dissolved in methanol. The standard and tested samples were filtered through a 0.22 μm (gold) filter and then analyzed by ultrahigh-performance liquid chromatography (UPLC). The analysis was carried out on a Waters HSS T3 liquid chromatography system, which consisted of a vacuum degasser, quaternary pump, automatic sampler and diode array detector (DAD). The DAD was connected with Waters ChemStation software. An Acquity UPLC H-class C18 column (2.1× 50 mm, inner diameter [ID] 1.7 μm) was used. Using acetonitrile and ultrapure water as eluents, the ginsenosides were separated by gradient elution. The flow rate was maintained at 0.3 mL·min^−1^. The injection volume was 3 μL, and the chromatogram was detected at 203 nm [25].

#### 2.6.4. Analysis of Monosaccharide

Complete acid hydrolysis: First, 2 mg of IRS was weighed, and then 0.5 mL of anhydrous methanol solution containing 2 mol/L HCl was added. The tube was sealed with N_2_ and the sample was hydrolyzed at 80 °C for 16 h and then dried with air. Next, 0.5 mL of 2 mol/L trifluoroacetic acid was added, and the sample was hydrolyzed for 1 h at 120 °C and then transferred to an evaporating dish in a water bath at 45 °C. Absolute ethanol was added repeatedly to remove the trifluoroacetic acid, and then the sample was dried.

Derivatization of 1-phenyl-3-methyl-5-pyrazolone (PMP): First, 0.5 mL of PMP reagent and 0.3 mol/L NaOH solution were added to the monosaccharide sample after hydrolysis, and 0.3 mL of PMP was put into a 5 mL centrifuge tube after full dissolution and allowed to react for 30 min at 70 °C in a water bath. After centrifugation (5000 rpm, 5 min), 0.3 mL of 0.3 mol/L HCl was added and mixed. Then, 2 mL of chloroform was added 3 times, the excess PMP reagent was extracted, the chloroform layer was removed by centrifugation (8000 rpm, 5 min), the water layer was filtered with a 0.22 μm (golden) filter and the samples were analyzed with high-performance liquid chromatography (HPLC).

HPLC analysis of monosaccharide-PMP derivatives: The mobile phase was 0.1 mol/L PB buffer (pH = 7.0): acetonitrile at 82:18 (*v/v*). The flow rate was 1.0 mL·min^−1^, the injection volume was 10 μL, and the detection wavelength was 245 nm [26].

#### 2.6.5. Analysis of Amino Acids

Establishment of a standard curve: A mixed standard solution of amino acids was prepared, and the concentration of each component was 2.5 mmol/L. The mixed standard solution was diluted with a 0.1 mol/L HCl solution, and the volume was fixed. An appropriate amount was placed into the sample bottle for testing. The VIS 1 wavelength was set to 570 nm, and VIS 2 was set to 440 nm.

Preparation of hydrolyzed amino acid samples: First, 100 mg of IRS was accurately weighed into a 50 mL hydrolysis tube, 20 mL of 6 mol/L hydrochloric acid solution was added and the tube was sealed with N_2_. The hydrolysis tube was hydrolyzed in a constant-temperature drying oven at 110 ℃ for 22 h. After cooling to room temperature, ultrapure water was used to fix the volume to 50 mL, 2 mL of the liquid was removed and the sample was evaporated to dryness in a vacuum drying oven at 70 ℃. Then, the residue was rinsed with ultrapure water of the same volume and evaporated twice, and 2 mL of buffer (0.02 mol/L HCl) was added for dilution. The sample was shaken well and filtered with a 0.22 μm (golden) filter into a sample bottle, where it was analyzed with an L-8900 amino acid automatic analyzer (Hitachi Company, Tokyo, Japan).

#### 2.6.6. Mineral Analysis

Al, Ni, Mn, Cu and Zn were determined by inductively coupled plasma mass spectrometry (ICP-MS) (Perkin Elmer Company, Waltham, Massachusetts, USA), and other elements were determined by flame atomic absorption spectrometry (FLAA) (Analytik Jena, Jena, Germany). The 0.5 mL liquid sample and 0.1 g solid sample were put into an acid mixture of 2.5 mL of HClO_4_ and 10 mL of HNO_3_ for digestion and then diluted with distilled water to 100 mL.

### 2.7. SEM and EDS Analyses

The microstructure of the sediments was observed by scanning electron microscope (SEM) (FEI Company TM, XL-30 ESEM FEG, Hillsboro, OR, USA). The samples were sprayed with gold-palladium alloy (Cressington Scientific Instruments Ltd., Watford, UK). The scanning images were captured at accelerating voltages of 15 kV and 20 kV with magnifications of 500× and 1000× (scale of 20 and 50 μm).

X-MAX energy dispersive spectrometry (EDS) was used for qualitative and quantitative analysis of elements in the sediments (Oxford Instruments, UK).

### 2.8. FT-IR and XRD Analysis

Fourier transform infrared spectroscopy (FT-IR) analyses of IRS were performed using a VERTEX 70 FT-IR spectrometer (Bruker Optics Inc., Ettlingen, Germany). The sample was mixed with potassium bromide powder in a ratio of 1:100 (*w*/*w*), and the spectra were obtained over the range of 4000–400 cm^−1^ with a resolution of 2 cm^−1^.

The structure of IRS was detected by X-ray diffractometry (D8 ADVANCE, Bruker AXS GmbH Co, Karlsruhe, Baden-Württemberg, Germany) equipped with theta-compensating slits and a Cu-Kα radiation source running at 30 mA and 40 kV. The diffraction angle (2θ) was adjusted from 5° to 90° at a rate of 2°/min. The crystallinity index (CI, %) of IRS was calculated by MDI Jade 6.0 software.

### 2.9. Establishment of Chemical Composition Interaction Model

Pectin (20 mg/mL), protein (4 mg/mL), proline (0.25 mg/mL), aspartic acid (0.5 mg/mL), glutamate (0.5 mg/mL), oxalic acid (0.1 mg/mL) and citric acid (0.1 mg/mL) were mixed with Ca^2+^ solution (CaCl_2_, 50 mg/L) at 1:1 volume ratio, respectively. Pectin, dextran (20 mg/mL), oxalic acid and citric acid were mixed with protein at 1:1 volume ratio, respectively. The mixed solution was subjected to complexation simulation test at 4 °C, 25 °C and 80 °C; the reaction time was 60 min. Then, the turbidity was measured by WZB-172 turbidimeter (Shanghai INESA Scientific Instrument Co., Ltd., Shanghai, China).

### 2.10. Data Analysis

All the experiments were repeated three times, and the data were expressed as average values. SPSS statistical software was used to conduct one-way ANOVA and Duncan multiple-range tests to determine the significant differences between the mean and variables, and GraphPad Prism 6.0 was used to draw the chart.

## 3. Results and Discussion

### 3.1. Observations of Sediment during Storage

**Figure 1:**
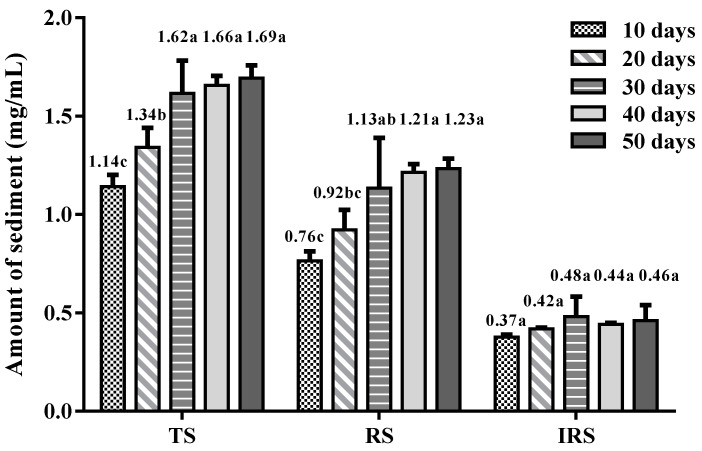
Total sediment (TS), reversible sediment (RS) and irreversible sediment (IRS) of ginseng extract during storage at 4 °C. The data are means of three replicates. Different letters in the column groups indicate a significant difference (*p* < 0.05).

The sediments from ginseng extract stored at low temperature (4 °C) for 0–50 days were analyzed. The amount of sediment increased during storage (Table 1). A small amount of sediment was visible to the naked eye starting on the second day. With an increase in storage days, the sediment in the ginseng extract continued to form and exhibited stratification. On the 10th day, most of the yellow sediment was produced, and white sediment began to form. From the 20th to the 30th day, the amount of yellow sediment increased continuously, while the amount of white sediment increased obviously. From the 30th to the 40th day, the amounts of both the yellow sediment and the white sediment increased slightly. From the 40th to the 50th day, the amounts of yellow sediment and white sediment did not increase. According to the sediment analysis (Figure 1), the total sediment showed an increasing trend from day 0 to day 50, increased significantly in the first 30 days and tended to be stable on days 30–50. RS (67.5–73.5%) was the principal part of the sediment, which played a dominant role in the change in total sediment. The proportion of IRS (26.5–32.5%) was relatively small, and there was no significant change during storage. It was observed that most of the yellow sediment can be redissolved (70 °C, 20 min), while the white sediment is almost insoluble.

### 3.2. Chemical Composition Analysis of the Extract and Sediments

During low-temperature storage, the chemical components of the ginseng supernatant changed to different degrees (Table 2). With increased time, in addition to Na, the elements K, Mg, Ca, Al, Fe, Mn, Ni, Sr and Ba all showed a decreasing trend. There is no significant difference in Mg and Mn. The highest content of K in the ginseng supernatant was 85-88% of the total metal elements. Most elements began to decline significantly on the 10th day. Contents of free amino acids, proteins, total sugar and total saponins did not change significantly during storage. The highest content in the supernatant was total sugar, which accounted for 71–74% of the main chemical components. Except Re, the content of ginsenosides changed significantly with the extension of storage time: the contents of Rg_1_, Rb_1_, Rc, Rb_2_, Rb_3_ and Rd decreased significantly, and Rf showed an increasing trend. The total contents of the 8 ginsenosides also showed a significant downward trend. The results showed that sediment formation was the main reason for the decrease in chemical components in the supernatant.

The data are the means of three replicates. Different letters on the same row in each group indicate significant differences between mean values (*p* < 0.05). Data > 10 take integers, data between 1–10 keep 1 significant decimal place and data < 1 keep 3 significant decimal places. Most of the RS in the ginseng extract was yellow sediment. Table 2 shows that there was no significant difference in the metal elements in RS during storage. Except for K and Mg, other elements, such as Na, Ca, Al, Fe, Mn, Ni, Sr and Ba, showed a downward trend. In addition, K was the element with the highest content in the RS, indicating that K participates to a higher degree in the RS. Free amino acids, protein and total saponins increased significantly with storage time, while total sugar content did not change significantly. According to the content, carbohydrates are the main component of RS, followed by proteins, total saponins and free amino acids. The contents of Rg_1_, Re, Rb_1_, Rc, Rb_2_ and total contents of ginsenosides increased significantly.

The key to removing the sediment from the ginseng extract is to inhibit the formation of IRS. We analyzed the metal elements in the IRS (Table 2). The elements increased significantly with the extension of storage time. K, Mg, Na, Ca, Al and Fe were the main elements in the IRS, and the content of Ca was the highest, accounting for 73–76% of the total metal elements, followed by K (9–16%) and Al (5–8%). During the 50 days of investigation, the contents of K, Al and Ni increased more than 2 times, and the Ca content increased more than 4 times. This shows that a large number of metal elements participate in the formation of IRS.

### 3.3. SEM and EDS Analysis

**Figure 2:**
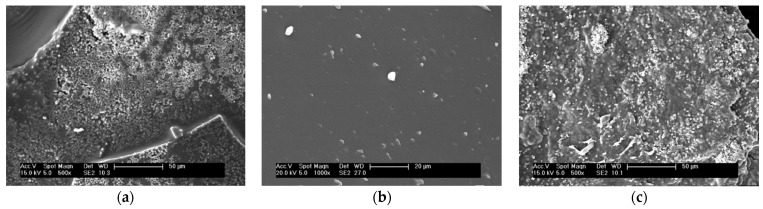
Scanning electron microscopy (SEM) images of white sediment (**a**), yellow sediment (**b**) and irreversible sediment (**c**).

In order to investigate the characteristics of white sediment (WS) and yellow sediment (YS), they were separated manually and scanned by electron microscopy (Figure 2). The results show that there are dense crystal structures in the white sediment (Figure 2a); The structure of yellow sediment is smooth without crystal structure (Figure 2b). It can be clearly seen that many encapsulated crystals (Figure 2c) are also present in the IRS. The IRS is composed of white sediment and yellow sediment. This crystal structure also exists in wine, which has been proved to be tartrate [16], and there is similar organic acid salt crystal in barberry juice [27], which is a common phenomenon in fruit juice or fruit wine. The above results indicated that the combination of metal ions and organic acids was involved in the formation of sediment during the storage process of ginseng extract, and most of the white sediment were metal salts. Through further energy spectrum analysis, the content of Ca in the WS was determined to be 8.72%, which is significantly higher than that in the yellow sediment (0.17%) and IRS (5.32%). It can be inferred that the crystals in the white sediment are mainly Ca^2+^. In the yellow sediment, C and O accounted for 97.45%, K accounted for 1.6% and other elements accounted for a small amount. This result indicates that the main component of yellow sediment was carbohydrates, and some sylvite was formed.

### 3.4. FT-IR and XRD Analyses

**Figure 3:**
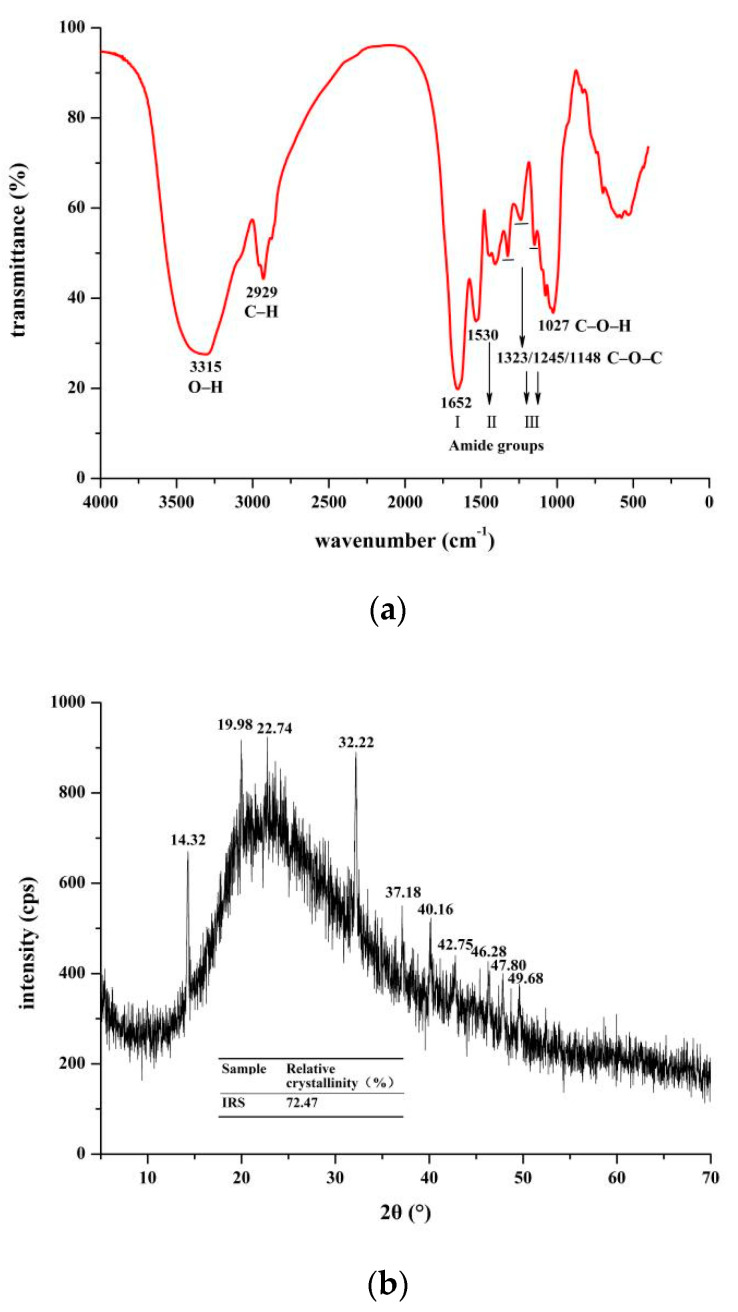
Fourier transform infrared spectroscopy (FT-IR, (**a**)) and X-ray diffractometry (XRD, (**b**)) results of IRS. The relative crystallinity was calculated by MDI Jade 6.0.

The main functional groups in the IRS of ginseng water extract were determined by FT-IR spectroscopy (Figure 3a). The FT-IR spectrum of IRS shows typical absorption peaks for some specific groups. The peak at 3315 cm^−1^ was attributed to the stretching vibration of O-H; the absorption peak here is wider, indicating that there are more hydrogen bonds in the association state [28]. The peak at 2929 cm^−1^ was attributed to the C–H stretching vibration of carbohydrate methyl and methylene. The above absorption peaks are characteristic of polysaccharides [29]. The absorption bands between 1652 cm^−1^ and 1530 cm^−1^ were related to amide I (C=O stretching) and amide II bands (N–H stretching), respectively [30], which indicated the presence of uronic acid and protein in IRS. The peaks at 1323 cm^−1^ and 1245 cm^−1^ of IRS were attributed to the amide III band (C–N stretching). The secondary structure of the protein in IRS was determined to be an α-helix structure by combining the amide I and III bands. The absorption peaks at 1148 cm^−1^ and 1027 cm^−1^, which are attributed to the stretching vibrations of C–O–C and C–O–H groups, indicate that pyran rings exist in IRS [31]. In addition, it was found that the IRS had a spectral peak very similar to that of the high-fat pectin-pea protein isolate complex [32]. In conclusion, the IRS had functional groups typical of polysaccharides and proteins, which might be caused by amidation between the amino groups of protein molecules and the carboxyl groups of pectin chains or by the production of insoluble components by protein molecules and polysaccharides produced under the action of static electricity.

The crystal structure of IRS was analyzed by X-ray diffractometry. The XRD spectrum showed several obvious diffraction peaks (Figure 3a), which indicated that the IRS had a crystal structure consistent with the results observed by SEM (Figure 2c). The spectrum shows strong diffraction peaks at 2θ = 14.32°, 19.98°, 22.74° and 32.22° and weaker diffraction peaks at 37.18–49.68°. It has a profile similar to that of the calcium oxalate dihydrate (COD) crystals obtained via electrocrystallization of indium oxide modified with R-chitosan fibers [33]. In addition, COD has been proved to be more regular than COM in crystal structure, showing a quadrilateral with blunt edges [27,34]. In Figure 2a, we observed similar structures, and some organic acids can also participate in sediment at low concentrations [19]. At the same time, the main diffraction peak was consistent with JCPDS card number 20-0233 (COD). The crystallinity of the IRS calculated by Jade 6.0 software was 72.47%.

### 3.5. Monosaccharide and Amino Acid Composition Analysis of IRS

**Figure 4:**
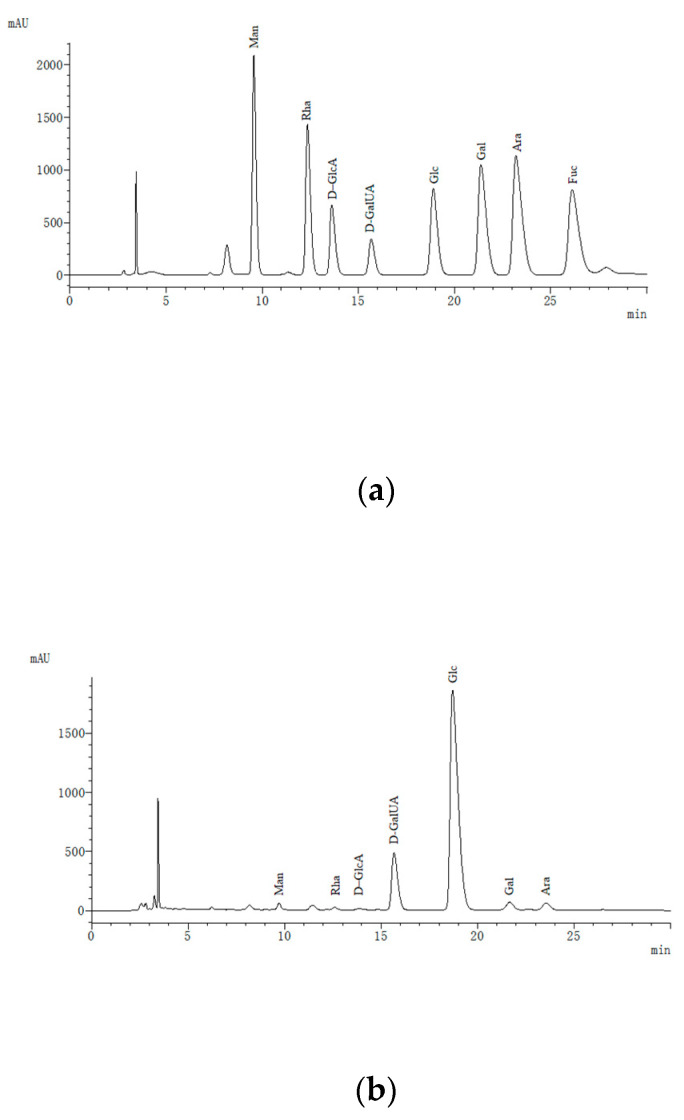

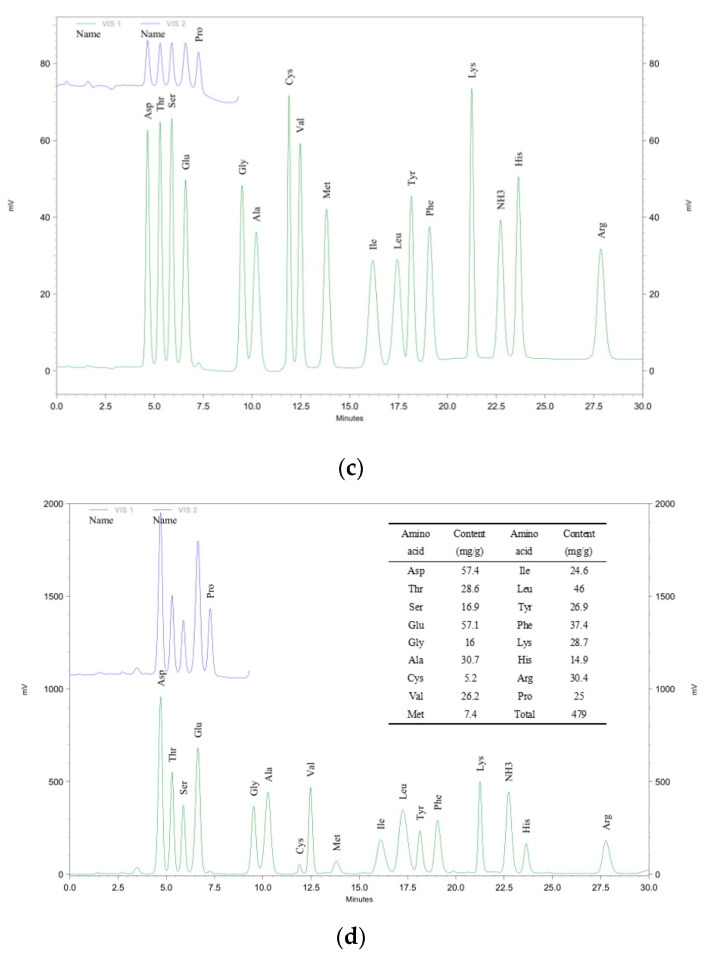
Composition of monosaccharides and amino acids in the IRS ((**a**): monosaccharide content, (**b**): monosaccharide composition in the IRS, (**c**): amino acid content, (**d**): amino acid composition in the IRS). Man: mannose; Rha: rhamnose; d-GlcA: d-gluconic acid; d-GalUA: d-galacturonic acid; Glc: glucose; Gal: galactose; Ara: arabinose; Fuc: fucose. Asp: aspartate; Thr: threonine; Ser: serine; Glu: glutamic; Gly: glycine; Ala: alanine; Cys: cysteine; Val: valine; Met: methionine; Ile: isoleucine; Leu: leucine; Tyr: tyrosine; Phe: phenylalanine; Lys: lysine; His: histidine; Arg: arginine; Pro: proline. Data are the means of three replicates.

The IRS was hydrolyzed to detect 8 monosaccharide components. The results showed that the IRS contained 7 monosaccharides (Figure 4b), namely mannose (Man), rhamnose (Rha), D-gluconic acid (D-GluA), D-galacturonic acid (D-GalUA), glucose (Glu), galactose (Gal) and arabinose (Ara). Glucose was the main monosaccharide in the IRS, followed by D-galacturonic acid, and the remaining monosaccharides accounted for a small proportion. In addition, 17 amino acids were detected (Figure 4d). Proline was observed in channel 2, and the remaining amino acids were observed in channel 1. The total amino acid content in the IRS was 479 mg/g. The contents of aspartate (Asp, 11.98%), glutamic acid (Glu, 11.92%) and leucine (Leu, 9.60%) were relatively high. The charge effect of polysaccharides and proteins was an important factor in inducing the transformation of polysaccharide-protein systems, and the polymerization of gel particles was positively correlated with the density of charge [14,35]. Protein and pectin easily bind together, and β-lactoglobulin can interact with at least 7 adjacent D-galacturonic acids, indicating that protein–pectin binding is closely related to the length of the pectin chain [36,37]. In addition, aspartate and glutamic acid in the IRS are acidic amino acids, and during the storage of the ginseng extract, these acidic amino acids can form insoluble amino acid salts with metal ions such as Ca [38,39]. Leucine is a nonpolar amino acid with a hydrophobic side chain that has low solubility in water and easily precipitates. Ca^2+^ can combine with low-degree esterified pectin to form a stable egg-box structure [40,41]. This structure plays an important role in determining cell wall biomechanics and mediating cell adhesion and has a wide range of applications in the field of material engineering [42,43].

### 3.6. Verification of Chemical Composition Interaction

**Figure 5:**
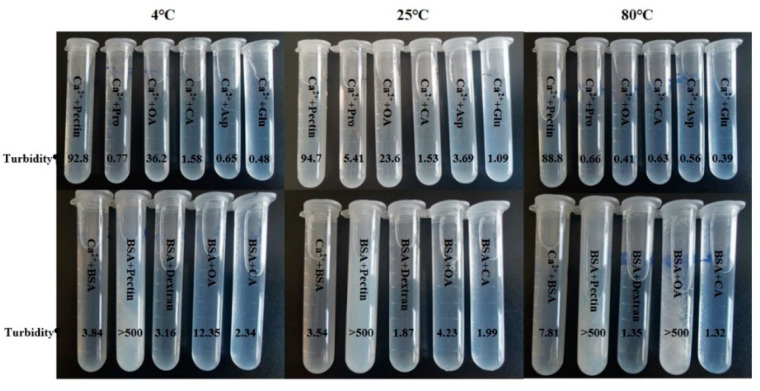
Turbidity of chemical composition interaction at different temperatures.

The formation of complexes in solution is mainly affected by pH, ionic strength, conformation, charge density, protein and polysaccharide concentration [39]. Based on the above results, the main chemical component interaction model system was established at 4 °C, 25 °C and 80 °C, respectively, to observe the turbidity formation of the component interaction and determine the turbidity (Figure 5). The results showed that the turbidity of Ca^2+^ combined with pectin was significantly higher than when combined with other components, which verified that the complexation of Ca^2+^ with pectin was one of the reasons for the turbidity of the ginseng extract. The turbidity of Ca^2+^ and oxalic acid decreases with the increase of reaction temperature, which may be due to the decarboxylation reaction of oxalic acid during the heating process. In addition, under the three reaction conditions, the combination of protein and pectin is the most turbid and produces colloids. As we all know, the interaction between protein and polysaccharide is a common model of component interaction. It is widely used in the fields of food emulsifiers and bioactive substance delivery and plays a crucial role in the structure and stability of food [44]. Under electrostatic action (with opposite charged groups), complex condensation or binding phase separation occurs, resulting in the formation of protein–polysaccharide complexes [45]. The pectin with higher local charge density has a stronger affinity for the binding site, so it is easier to complex aggregation with proteins, and high-charge polysaccharides are more conducive to the formation of precipitation [46]. The mixture of protein and oxalic acid at 25 °C and 4 °C did not appear turbid, but the mixture appeared with flocculent precipitation at 80 °C. The high temperature caused protein denaturation, and then the secondary bonds were destroyed, hydrophobic bonds in the protein are exposed and precipitate.

## 4. Conclusions

The purpose of this paper was to elucidate the formation mechanism of sediment in a ginseng extract during low-temperature storage. The sediment formation was clarified by observing the ginseng extract at low temperature for 50 days. The observations showed that the sediment was stratified, with yellow and white portions. The first 30 days of storage was the critical period for sediment formation, and after 30 days, the amount of sediment entered a stable period.

The chemical composition analysis of the extract and sediment showed that a large number of metal elements were involved in the formation of the sediment. The increase in Ca in the IRS was the most significant, which was more than 4 times. The SEM and EDS results also confirmed the existence of a large amount of Ca, and the IRS was composed of white and yellow sediments. The FT-IR spectrum and X-ray diffraction results showed that there were protein and polysaccharide complex in the IRS, and the crystal structure was consistent with COD. Seven monosaccharides and 17 amino acids were detected in the IRS. Glucose and galacturonic acid were the main monosaccharides, and aspartate and glutamic acid were the main amino acids.

The interaction model of the key chemical components at the three reaction temperatures showed that the protein and pectin had a complex reaction due to electrostatic interaction, resulting in the greatest degree of turbidity and a colloidal structure. Secondly, Ca^2+^ and pectin form the egg-box structure will cause the liquid turbidity. The turbidity decreases after the high temperature reaction of Ca^2+^ and oxalic acid, it may be caused by the decarboxylation reaction of oxalic acid. Due to high temperature denaturation of protein, the turbidity of the mixture of protein and oxalic acid is much higher than that of normal and low temperature.

The above results indicated that the sediment of ginseng extract was formed by the physical factors and interaction of various chemical components. Regulation of IRS is the key to solve the sediment of ginseng extract. The stable state system can be improved by regulating Ca^2+^ complexation, changing protein and polysaccharide structures and increasing liquid viscosity. This study has practical significance for improving the quality and economic benefit of ginseng beverage.

## Figures and Tables

**Table 1:** Observations of ginseng extract sediment formation during storage at 4 °C.

Storage (Days)	Observation	Amount of Sediments		Description
		Yellow Sediments	White Sediments	
0	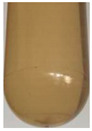	No	No	The extract was clarified without sediment.
10	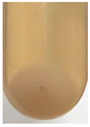	Some	Little	Some quantity of yellow sediments and a small quantity of white sediment appeared.
20	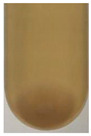	Much	Little	Compared to the sediment stored for 10 days, the yellow sediment increased significantly, and the white sediment did not change significantly.
30	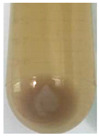	Much	Some	Compared to the sediment stored for 20 days, both the yellow sediment and the white sediment increased significantly.
40	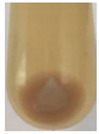	Much	Some	Compared to the sediment stored for 30 days, both the yellow sediment and the white sediment increased but not significantly.
50	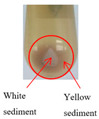	Much	Some	Compared to the sediment stored for 40 days, both the yellow sediment and the white sediment increased only slightly.

The pictures show the sediment after centrifugation.

**Table 2:** Chemical composition analysis of ginseng supernatant, RS and IRS.

Chemical Components		Supernatant						RS					IRS				
		0 Day	10 Day	20 Day	30 Day	40 Day	50 Day	10 Day	20 Day	30 Day	40 Day	50 Day	10 Day	20 Day	30 Day	40 Day	50 Day
K	mg/kg	1088 ^a^	1080 ^a^	1032 ^b^	1032 ^b^	1024 ^c^	1014 ^c^	31 ^c^	33 ^c^	39 ^b^	49 ^a^	51 ^a^	1551 ^b^	1706 ^b^	3672 ^a^	3236 ^a^	3614 ^a^
Mg		86 ^a^	85 ^a^	84 ^a^	82 ^a^	81 ^a^	80 ^a^	2.9 ^c^	3.6 ^c^	4.3 ^b^	4.9 ^ab^	5.1 ^a^	625 ^b^	630 ^b^	680 ^ab^	704 ^a^	735 ^a^
Na		19 ^b^	18 ^b^	20 ^a b^	20 ^ab^	21 ^a b^	22 ^a^	11 ^a^	9.2 ^ab^	7.4 ^b^	6.5 ^b^	4.4 ^c^	357 ^c^	376 ^c^	412 ^bc^	536 ^ab^	601 ^a^
Ca		47 ^a^	31 ^b^	29 ^b^	28 ^b^	26 ^b^	25 ^b^	9.1 ^a b^	11 ^a^	11 ^a^	10 ^a^	8.7 ^b^	6224 ^d^	14678 ^c^	16186 ^c^	20137 ^b^	25024 ^a^
Al		2.8 ^a^	2.2 ^b^	2.0 ^b^	1.9 ^b^	1.4 ^b^	1.3 ^b^	2.3 ^a^	2.2 ^a^	1.6 ^b^	1.1 ^b c^	0.800 ^cd^	771 ^e^	964 ^d^	1210 ^c^	1738 ^b^	2048 ^a^
Fe		2.6 ^a^	2.3 ^b^	2.2 ^b c^	2.0 ^cd^	2.0 ^c d^	1.9 ^e^	0.633 ^a^	0.621 ^a^	0.616 ^a^	0.446 ^b^	0.411 ^b^	213 ^c^	220 ^c^	224 ^c^	261 ^b^	312 ^a^
Mn		20 ^a^	20 ^a^	19 ^a b^	19 ^ab^	19 ^a b^	19 ^a b^	0.234 ^a^	0.207 ^b^	0.200 ^b^	0.187 ^bc^	0.165 ^c^	50 ^d^	52 ^d^	58 ^c^	70 ^b^	74 ^a^
Ni		0.150 ^a^	0.145 ^a b^	0.141 ^b^	0.139 ^b^	0.133 ^c^	0.133 ^c^	0.018 ^a^	0.013 ^b^	0.012 ^b^	0.012 ^b^	0.011 ^b^	1.1 ^e^	1.5 ^d^	1.8 ^c^	2.1 ^b^	2.4 ^a^
Sr		0.248 ^a^	0.221 ^a b^	0.210 ^b^	0.219 ^b^	0.203 ^b^	0.198 ^b^	0.033 ^a^	0.031 ^a^	0.029 ^ab^	0.024 ^bc^	0.022 ^c^	33 ^d^	39 ^c^	40 ^c^	45 ^b^	53 ^a^
Ba		0.054 ^a^	0.049 ^a b^	0.048 ^b^	0.045 ^b^	0.045 ^b^	0.044 ^b^	0.007 ^a^	0.007 ^a^	0.006 ^ab^	0.005 ^ab^	0.003 ^b^	15 ^c^	16 ^b^	16 ^b^	20 ^a^	20 ^a^
Free amino acid	mg/mL	5.3 ^a^	5.6 ^a^	5.5 ^a^	5.8 ^a^	5.8 ^a^	5.8 ^a^	0.068 ^b^	0.069 ^b^	0.102 ^a^	0.106 ^a^	0.110 ^a^					
Protein		5.9 ^a^	5.5 ^a^	5.4 ^a^	5.1 ^a^	5.0 ^a^	5.0 ^a^	0.282 ^d^	0.333 ^cd^	0.374 ^bc^	0.436 ^ab^	0.506 ^a^					
Total sugar		44 ^a^	44 ^a^	43 ^a^	42 ^a^	41 ^a^	40 ^a^	0.881 ^a^	1.0 ^a^	1.5 ^a^	1.5 ^a^	1.7 ^a^					
Total saponins		4.1 ^a^	3.8 ^a b^	4.0 ^a^	3.8 ^ab^	3.7 ^ab^	3.7 ^ab^	0.141 ^b^	0.156 ^ab^	0.165 ^a b^	0.168 ^ab^	0.185 ^a^					
Rg1		0.409 ^a^	0.402 ^b^	0.397 ^c^	0.396 ^cd^	0.388 ^e^	0.392 ^d^	0.007 ^c^	0.012 ^b^	0.011 ^b^	0.015 ^a^	0.017 ^a^					
Re		0.211 ^a^	0.306 ^a^	0.305 ^a^	0.307 ^a^	0.296 ^a^	0.291 ^a^	0.004 ^c^	0.008 ^b^	0.008 ^b^	0.012 ^a^	0.012 ^a^					
Rf		0.217 ^c^	0.224 ^c^	0.150 ^d^	0.221 ^c^	0.422 ^a^	0.383 ^b^	0.003 ^c^	0.005 ^b^	0.005 ^b^	0.006 ^a^	0.005 ^b^					
Rb1		0.769 ^a^	0.743 ^b^	0.737 ^c^	0.737 ^c^	0.618 ^d^	0.454 ^e^	0.010 ^d^	0.009 ^d^	0.023 ^c^	0.024 ^b^	0.031 ^a^					
Rc		0.285 ^a^	0.276 ^b^	0.266 ^c^	0.243 ^d^	0.231 ^e^	0.242 ^d^	0.004 ^d^	0.008 ^c^	0.010 ^b^	0.010 ^b^	0.012 ^a^					
Rb2		0.325 ^a^	0.295 ^c^	0.307 ^b^	0.280 ^d^	0.272 ^e^	0.188 ^f^	0.004 ^d^	0.009 ^c^	0.009 ^c^	0.011 ^b^	0.012 ^a^					
Rb3		0.038 ^a^	0.032 ^b^	0.028 ^c^	0.028 ^c^	0.022 ^d^	0.012 ^e^	0.001 ^a^	0.001 ^a^	0.001 ^a^	0.001 ^a^	0.001 ^a^					
Rd		0.079 ^a^	0.075 ^b^	0.069 ^c^	0.052 ^d^	0.046 ^e^	0.041 ^f^	0.001 ^b^	0.001 ^b^	0.001 ^b^	0.002 ^a^	0.001 ^b^					
Total ginsenoside		2.9 ^ab^	3.0 ^a^	2.8 ^bc^	2.8 ^bc^	2.7 ^c^	2.4 ^d^	0.034 ^e^	0.053 ^d^	0.072 ^c^	0.087 ^b^	0.093 ^a^					

Different letters in the column groups indicate a significant difference (*p* < 0.05).
